# Palmitate induces ER calcium depletion and apoptosis in mouse podocytes subsequent to mitochondrial oxidative stress

**DOI:** 10.1038/cddis.2015.331

**Published:** 2015-11-19

**Authors:** S Xu, S M Nam, J-H Kim, R Das, S-K Choi, T T Nguyen, X Quan, S J Choi, C H Chung, E Y Lee, I-K Lee, A Wiederkehr, C B Wollheim, S-K Cha, K-S Park

**Affiliations:** 1 Department of Physiology and Institute of Lifestyle Medicine, Yonsei University Wonju College of Medicine, Wonju, Korea; 2 Department of Internal Medicine, Daejeon Sun Hospital, Daejeon, Korea; 3 Department of Microbiology, Yonsei University Wonju College of Medicine, Wonju, Korea; 4 Department of Internal Medicine, Yonsei University Wonju College of Medicine, Wonju, Korea; 5 Department of Internal Medicine, Soonchunhyang University Cheonan Hospital, Cheonan, Korea; 6 Department of Internal Medicine, Kyungpook National University Hospital, Daegu, Korea; 7 Nestlé Institute of Health Sciences, Lausanne, Switzerland; 8 Department of Cell Physiology and Metabolism, University of Geneva, Geneva, Switzerland

## Abstract

Pathologic alterations in podocytes lead to failure of an essential component of the glomerular filtration barrier and proteinuria in chronic kidney diseases. Elevated levels of saturated free fatty acid (FFA) are harmful to various tissues, implemented in the progression of diabetes and its complications such as proteinuria in diabetic nephropathy. Here, we investigated the molecular mechanism of palmitate cytotoxicity in cultured mouse podocytes. Incubation with palmitate dose-dependently increased cytosolic and mitochondrial reactive oxygen species, depolarized the mitochondrial membrane potential, impaired ATP synthesis and elicited apoptotic cell death. Palmitate not only evoked mitochondrial fragmentation but also caused marked dilation of the endoplasmic reticulum (ER). Consistently, palmitate upregulated ER stress proteins, oligomerized stromal interaction molecule 1 (STIM1) in the subplasmalemmal ER membrane, abolished the cyclopiazonic acid-induced cytosolic Ca^2+^ increase due to depletion of luminal ER Ca^2+^. Palmitate-induced ER Ca^2+^ depletion and cytotoxicity were blocked by a selective inhibitor of the fatty-acid transporter FAT/CD36. Loss of the ER Ca^2+^ pool induced by palmitate was reverted by the phospholipase C (PLC) inhibitor edelfosine. Palmitate-dependent activation of PLC was further demonstrated by following cytosolic translocation of the pleckstrin homology domain of PLC in palmitate-treated podocytes. An inhibitor of diacylglycerol (DAG) kinase, which elevates cytosolic DAG, strongly promoted ER Ca^2+^ depletion by low-dose palmitate. GF109203X, a PKC inhibitor, partially prevented palmitate-induced ER Ca^2+^ loss. Remarkably, the mitochondrial antioxidant mitoTEMPO inhibited palmitate-induced PLC activation, ER Ca^2+^ depletion and cytotoxicity. Palmitate elicited cytoskeletal changes in podocytes and increased albumin permeability, which was also blocked by mitoTEMPO. These data suggest that oxidative stress caused by saturated FFA leads to mitochondrial dysfunction and ER Ca^2+^ depletion through FAT/CD36 and PLC signaling, possibly contributing to podocyte injury.

Podocytes are the terminally differentiated visceral epithelial cells in the glomerular filtration barrier that have a critical role in conserving macromolecules in the plasma. Injury, cell death and detachment of podocytes lead to proteinuria, an early prognostic symptom of chronic kidney disease (CKD).^1^ Epidemiologic studies have shown that the majority of CDKs in patients are caused by glomerular disorders with diabetic nephropathy (DN).^2, 3^ One of the major pathogenic mediators in type 2 diabetes and its complications is dyslipidemia, resulting in high saturated free fatty-acid (FFA) concentrations. In normal adults, the total plasma FFA concentration is reported to be 200–600 *μ*M, and this value increases up to fourfold in type 2 diabetes.^4^ Palmitate is the most abundant saturated FFA in the plasma of humans and rodents accounting for ~25% of total fatty acids.^5, 6^

Saturated FFAs in the cytosol induce reactive oxygen species (ROS) production as observed in pancreatic *β*-cells, hepatic cells and skeletal muscle cells.^7, 8, 9^ In muscle cells, palmitate stimulates superoxide generation through the mitochondrial electron transport chain and NADPH oxidase activities.^10^ This oxidative stress leads to mitochondrial dysfunction, mitochondrial permeability transition (PT), cytochrome c release and apoptosis.^8, 11, 12^ The role of unsaturated FFA in this process has been controversial, but accumulating evidence suggests a protective function counteracting the cytotoxic effect of saturated FFA.^8, 13, 14^

Furthermore, palmitate elicits the unfolded protein response and endoplasmic reticulum (ER) stress-induced cell death in various cell types.^15, 16^ Particularly in pancreatic *β*-cells, ER stress by palmitate leads to defective insulin secretion contributing to the progression of type 2 diabetes.^17^ An explanation for the induction of ER stress may be that saturated FFA disrupts ER Ca^2+^ homeostasis in *β*-cells.^15, 18^ Depletion of ER Ca^2+^ impairs the function of ER-resident chaperones, resulting in the recruitment of the immunoglobulin heavy chain binding protein (BIP) into the lumen of the organelle.^19^ A series of ER stress responses are initiated by the release of BIP from sensors in the ER membrane including inositol-requiring protein 1, activating transcription factor 6 and PKR-like ER kinase (PERK). Downstream signaling cascades of these activators lead to translation attenuation, upregulation of ER chaperone expression, ER-associated protein degradation and apoptosis.^20^

The ER is highly sensitive to the redox status.^21^ In the ER lumen, protein folding by disulfide isomerase requires oxidation, which depends on hydrogen peroxide generated by ER oxidoreductase (ERO1).^22^ Accumulation of misfolded proteins in the ER lumen produces excessive ROS through ERO1-mediated oxidation.^23^ Moreover, the induction of C/EBP homology protein (CHOP) caused by PERK activation during ER stress also increases ERO1 expression, which further accelerates ROS generation.^24, 25^ Intriguingly, Ca^2+^ release from the ER is increased during ER stress, which is blocked by silencing of ERO1 or type 1 inositol (1,4,5)-trisphosphate (IP_3_) receptor.^23^ These findings imply that oxidative stress aggravates ER stress in a vicious cycle by reducing the Ca^2+^ level in the ER lumen.

Several reports have investigated the pathologic changes induced by palmitate in podocytes, pertinent to the development of proteinuria and the progression of glomerular disease. Palmitate inhibits insulin signaling and insulin-stimulated glucose uptake in human podocytes.^26^ Palmitate-induced apoptosis associated with ER stress was partially prevented by silencing of CHOP in mouse podocytes.^14^ Furthermore, palmitate-dependent cytotoxicity was attenuated by the co-treatment with mono-unsaturated FFA or the activation of stearoyl-CoA desaturase, which converts saturated to unsaturated FFA.^27^

In this study, we aimed to identify the molecular mechanisms linking FFA-induced superoxide production associated with mitochondrial dysfunction and ER stress in mouse podocytes. We clearly show that palmitate disrupts Ca^2+^ homeostasis in the cytosol and intracellular organelles, crucial for the development of ER stress and cytotoxicity in podocytes. We further demonstrate that mitochondrial oxidative stress acts upstream of palmitate-induced ER Ca^2+^ depletion and ER stress through phospholipase C (PLC) activation. These perturbations of cellular homeostasis in podocytes with altered cytoskeletal arrangement likely contribute to the deterioration of the filtration barrier function in DN.

## Results

**Figure 1:**
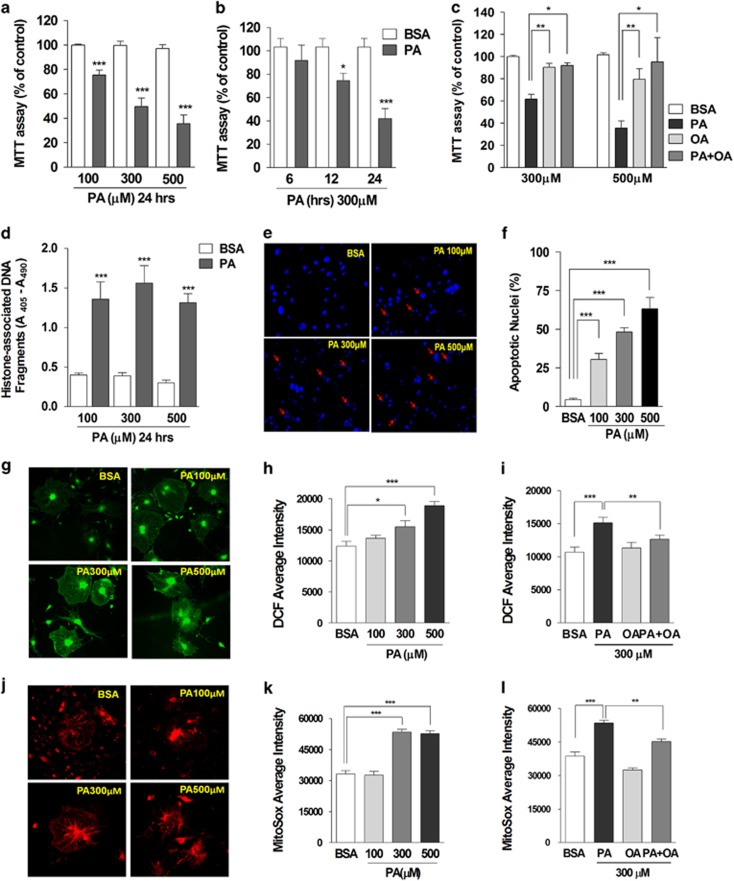
Palmitate, but not oleate, increased cytosolic and mitochondrial ROS production and induced cytotoxicity in mouse podocytes. Palmitate (PA) or bovine serum albumin (BSA) was applied to differentiated mouse podocytes with different concentrations ((**a**), *N*=10) and durations ((**b**), *N*=7). The effect of oleate (OA) on PA-induced cytotoxicity was also analyzed ((**c**), *N*=3). Cytotoxicity by palmitate treatment (24 h) was evaluated by MTT assay (**a**–**c**) and quantitative determination of apoptotic DNA fragments ((**d**), *N*=5). Condensed and fragmented apoptotic nuclei induced by palmitate were detected by DAPI staining (**e**) and are expressed as the percent of apoptotic nuclei ((**f**), *N*=8). Podocytes treated with BSA or palmitate (PA) were loaded with DCF-DA (**g**–**i**) or mitoSox (**j**–**l**) and the fluorescence intensity was measured using a fluorescence microscope imaging system. Average florescence intensities of DCF (19–29 images from ≥5 independent experiments; (**b**) and (**c**)) or mitoSox (7–10 images from ≥3 independent experiments; **e** and **f**) were analyzed. Data are presented as mean±S.E.M., and *, **, *** denote *P*<0.05, *P*<0.01 and *P*<0.001, respectively

We previously demonstrated full-podocyte differentiation after 14 days in culture at 37 °C without interferon-*γ*.^28^ To investigate the cytotoxic effects of FFA, we administered bovine serum albumin (BSA)-conjugated palmitate or oleate to differentiated podocytes. Using a 3-(4,5-dimethylhioazol-2-yl)-2,5-diphenyltetrazolium bromide (MTT) assay, we observed dose-dependent reduction of cell viability by 100–500 *μ*M palmitate (Figure 1a). Palmitate (300 *μ*M)-induced cytotoxicity was marked between 12 and 24 h of incubation (Figure 1b). Oleate did not affect cell survival and even protected from palmitate-induced cytotoxicity (Figure 1c). Incubation with palmitate for 24 h prominently raised apoptotic DNA fragments (Figure 1d). Palmitate dose-dependently increased the percentage of fragmented and condensed apoptotic nuclei (Figures 1e and f).

Cytosolic ROS generation was followed using the 2′,7′-Dichlorofluorescein diacetate (DCF-DA) fluorescence dye (Figures 1g–i). Treatment with palmitate for 24 h increased DCF fluorescence intensity. In contrast, oleate did not elevate cytosolic ROS. Moreover, palmitate-induced ROS generation was prevented by oleate co-incubation.

Mitochondrial superoxide was measured using mitoSox (Figures 1j–l). Palmitate treatment for 24 h increased the fluorescence intensity of mitoSox-loaded podocytes. Oleate did not affect mitochondrial ROS production, and attenuated the palmitate-induced increase of mitochondrial superoxide. These data demonstrate that the mono-unsaturated fatty-acid oleate protects from oxidative stress and cell death induced by saturated FFA in mouse podocytes.

**Figure 2:**
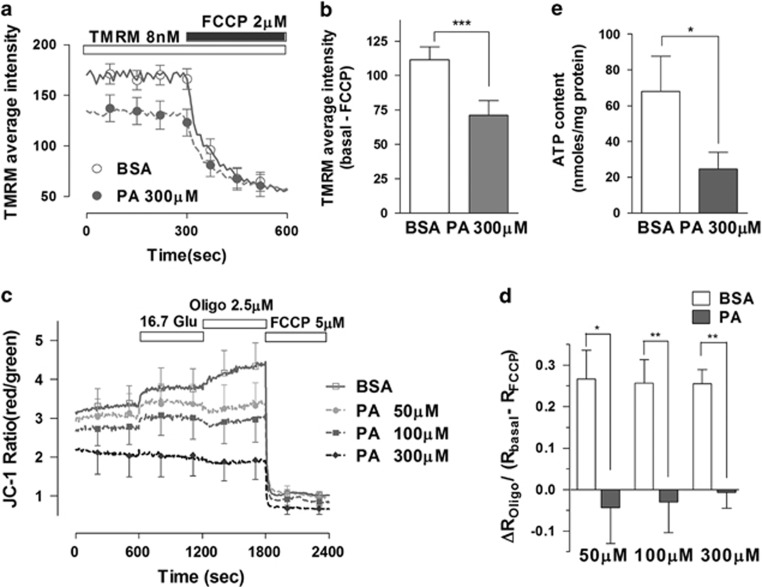
Palmitate elicited mitochondrial depolarization and ATP depletion in mouse podocytes. (**a**, **b**) Differentiated podocytes were loaded with the potential-sensitive probe TMRM (8 nM for 30 min) and changes in fluorescence intensity in the mitochondrial area were measured using a confocal microscope imaging system. Average TMRM fluorescence intensities were compared between BSA- and PA-treated podocytes (*N*=11). (**c**, **d**) Podocytes were loaded with JC-1 (350 nM for 30 min) and the fluorescence intensity was measured using a fluorescence multi-plate reader. The JC-1 fluorescence ratio of red over green, which reflects mitochondrial membrane potential, was recorded from BSA- or palmitate (PA)-treated cells (*N*=6–8). Oligomycin-induced changes in the JC-1 ratio (ΔR_Oligo_) were normalized to the resting mitochondrial membrane potential (R_basal_−R_FCCP_). (**e**) Podocytes were incubated with BSA or PA for 24 h and the cellular ATP content in podocytes was measured using a bioluminescence assay (*N*=13). Data are presented as mean±S.E.M., and *, ** and *** denote *P*<0.05, *P*<0.01 and *P*<0.001, respectively

We investigated whether palmitate incubation could alter the mitochondrial membrane potential (ΔΨm) and ATP generation as a consequence of mitochondrial oxidative stress. We used two potential-sensitive fluorescence dyes, tetramethylrhodamine methylester (TMRM) and JC-1 to measure the ΔΨm. TMRM (8 nM) was used in a non-quenching mode, distributing according to the Nernstian equation across the inner mitochondrial membrane. We observed that treatment with 300 *μ*M palmitate for 24 h lowered the average fluorescence intensity of TMRM in podocytes compared with control BSA-treated cells (Figures 2a and b).

As an alternative method to study ΔΨm, we measured the JC-1 fluorescence ratio (red/green) from BSA- or palmitate-treated podocytes (Figures 2c and d).^29^ The resting ΔΨm and glucose (16.7 mM)-stimulated hyperpolarization were lower in palmitate-treated cells. Oligomycin, a selective blocker of the F_1_F_0_-ATPase, causes hyperpolarization in normally functioning mitochondria, but depolarization in dysfunctional mitochondria.^30^ In control cells, oligomycin caused hyperpolarization but induced partial depolarization of the Ψm in podocytes treated with low concentration of palmitate. This finding suggests that palmitate treatment increases the fraction of impaired mitochondria consuming ATP through F_1_F_0_-ATPase to maintain the ΔΨm.

The lowered driving force across the inner mitochondrial membrane may also inhibit ATP synthesis. Incubation of podocytes with palmitate (300 *μ*M) for 24 h reduced the cellular ATP content compared with that of control cells (Figure 2e). Our results demonstrate palmitate-induced impairment of mitochondrial energy metabolism.

**Figure 3:**
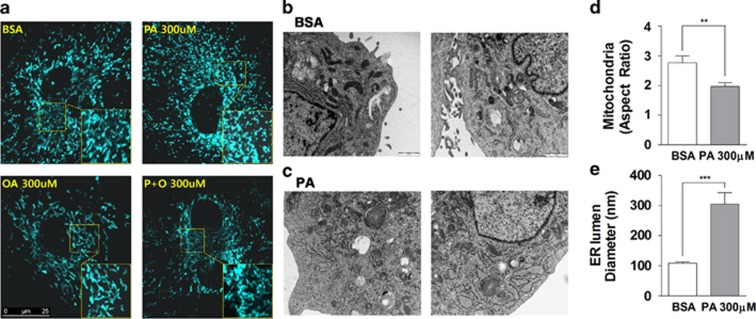
Palmitate-induced mitochondrial fragmentation and ER lumen dilation in mouse podocytes. (**a**) Differentiated podocytes were infected with adenovirus encoding mitochondrial-targeted cyan fluorescence protein (mitoCFP) and treated with BSA or palmitate (PA) for 24 h. Mitochondrial morphology was observed using a confocal microscope imaging system. (**b**, **c**) Structural alterations in intracellular organelles were analyzed from electron microscopy (EM) images. The aspect ratio of mitochondria (longer/shorter diameter, (**d**)) and the diameter of the ER lumen (**e**) were obtained from EM images and compared between BSA (*N*=13)- and PA (*N*=16)-treated podocytes. Data are presented as mean±S.E.M. ** and *** denote *P*<0.01 and *P*<0.001, respectively

Oxidative stress and depolarization of the Ψm has a negative impact on mitochondrial fusion resulting in shortened and fragmented mitochondria.^31, 32^ We observed that control podocytes had interconnected and rod-shaped mitochondria (Figure 3a). On the contrary, palmitate (300 *μ*M)-treated podocytes had dot-like round and fragmented mitochondria, suggesting either reduced mitochondrial fusion or accelerated fission. Oleate alone or added in combination with palmitate did not affect mitochondrial morphology.

Using electron microscopy (EM), we analyzed micro-structural alterations in intracellular organelles focusing on mitochondria and the ER (Figures 3b–e). BSA-treated cells had elongated mitochondria and regular width of ER lumen. On the other hand, shortened mitochondria and severely dilated ER was observed in palmitate-treated podocytes. We compared the mitochondrial aspect ratio (AR), which is calculated from the ratio of the longer diameter divided by the shorter one. Palmitate-treated podocytes had a lower mitochondrial AR value than BSA-treated cells. The mean ER diameter in palmitate-treated podocytes was conspicuously wider than the diameter in control cells, suggesting an ER stress response in palmitate-treated cells.^33^

**Figure 4:**
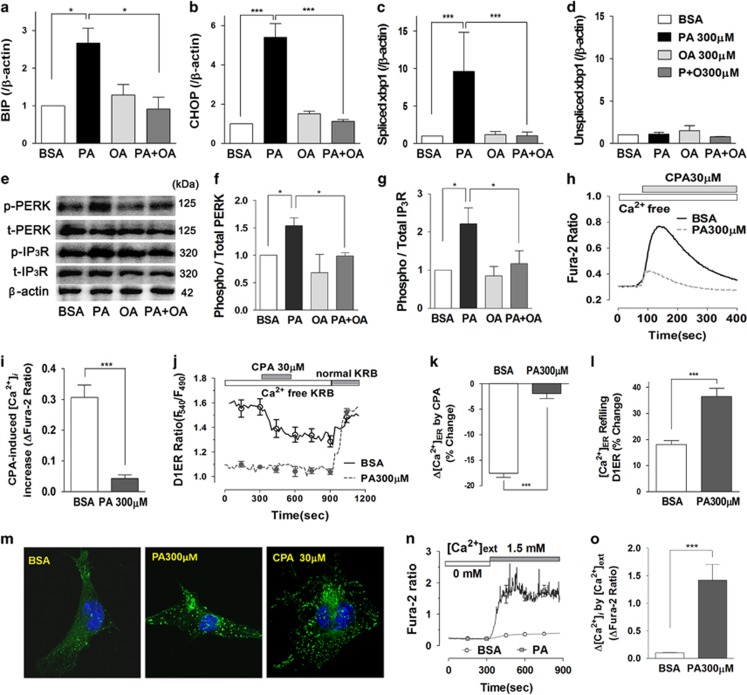
Palmitate depleted the ER Ca^2+^ pool and elicited ER stress in mouse podocytes. (**a**–**d**) Transcript levels of BIP (**a**), CHOP (**b**) and spliced (**c**) and unspliced xbp1 (**d**) were analyzed using quantitative RT-PCR from differentiated podocytes treated with BSA-, palmitate (PA)-, oleate (OA) and PA with OA for 24 h (*N*=3). (**e**–**g**) Protein levels of phosphorylated and total PKR-like ER kinase (PERK) and type 1 inositol (1,4,5)-trisphosphate receptor (IP_3_R) were analyzed using western blot. (**h**, **i**) Changes in cytosolic Ca^2+^ ([Ca^2+^]_i_) induced by cyclopiazonic acid (CPA) were recorded by using a fluorescence microscope imaging system, and reflect the amount of ER Ca^2+^ release. Before Fura-2 loading, podocytes were incubated with BSA or PA in Ca^2+^-free KRB solution for 90 min (*N*=8–9). (**j**–**l**) Luminal Ca^2+^ content in ER ([Ca^2+^]_ER_) was measured in podocytes transfected with plasmid encoding ER Ca^2+^-sensing fluorescence protein D1ER using a confocal microscope imaging system. CPA-induced reduction of [Ca^2+^]_ER_ and ER Ca^2+^ refilling by 1.8 mM extracellular Ca^2+^ were compared between podocytes treated with BSA (*N*=37) or PA (*N*=34) in Ca^2+^-free KRB solution for 90 min. (**m**) To visualize the localization of STIM1, podocytes were transfected with a plasmid encoding STIM1-yellow fluorescence protein (YFP). Confocal YFP fluorescence images were obtained from BSA-, PA- (for 24 h) or CPA- (for 10 min) treated podocytes. (**n**, **o**) Changes in [Ca^2+^]_i_ by extracellular Ca^2+^ addition were compared between BSA- or PA-treated podocytes in Ca^2+^-free KRB solution for 90 min (*N*=5). Data are presented as mean±S.E.M. * and *** denote *P*<0.05 and *P*<0.001, respectively

To investigate whether ER stress occurs in palmitate-treated podocytes, we checked transcriptional regulation of ER stress response genes (Figures 4a–d).^15^ Palmitate (300 *μ*M) increased mRNA levels of BIP and CHOP. Unspliced xbp1 (X-box BIP 1) was unaltered, whereas spliced xbp1 was highly upregulated by palmitate treatment. Oleate alone did not affect and in combination with palmitate prevented upregulation of ER stress markers. Palmitate increased phosphorylation of PERK and type 1 IP_3_ receptor (IP_3_R). These activations were prevented by oleate co-incubation (Figures 4e–g).

Because of the strong Ca^2+^ dependency of ER chaperones, ER Ca^2+^ depletion can cause misfolding of luminal proteins and as a consequence ER stress.^15, 18^ To estimate ER Ca^2+^ levels, we measured cytosolic Ca^2+^ ([Ca^2+^]_*i*_) changes in response to the application of cyclopiazonic acid (CPA), an inhibitor of the ER Ca^2+^ ATPase (SERCA) (Figures 4h and i). Podocytes were incubated with BSA or palmitate in Ca^2+^-free Krebs-Ringer bicarbonate (KRB) solution for 90 min. CPA caused a pronounced rise of [Ca^2+^]_i_ in control cells. This calcium rise was almost completely prevented when podocytes were treated with palmitate. The results reveal palmitate-induced loss of the ER Ca^2+^ store.

Next, we directly measured luminal Ca^2+^ level in the ER with D1ER (Figures 4j–l).^18, 34, 35^ In BSA-treated cells perfused with Ca^2+^-free KRB solution, the CPA-induced inhibition of the ER Ca^2+^ pump decreased the D1ER ratio. Palmitate-treated podocytes had a very low D1ER ratio at resting state, and did not significantly respond to CPA treatment. After wash out of CPA, the ER Ca^2+^ levels returned to basal level when exposed to 1.8 mM Ca^2+^. Following depletion of ER Ca^2+^ stores palmitate-treated podocytes showed more rapid and pronounced increases in ER Ca^2+^ refilling. These results demonstrate that store-operated Ca^2+^ entry (SOCE) and refilling of ER Ca^2+^ via the SERCA pump is not impaired following palmitate incubation.

SOCE is initiated by oligomerization of stromal interaction molecule 1 (STIM1) and Orai at the plasma membrane-ER junction area.^36^ Oligomerization in podocytes was followed in cells expressing yellow fluorescence protein (YFP)-tagged STIM1. Palmitate treatment-elicited redistribution of YFP into subplasmalemmal bright fluorescent spots suggestive of STIM1 oligomerization. A concentration of STIM1-YFP fluorescence was also observed in cells depleted for ER Ca^2+^ using CPA (Figure 4m). STIM1 oligomerization is consistent with palmitate-mediated ER Ca^2+^ depletion. To demonstrate the activation of SOCE by palmitate, we applied extracellular Ca^2+^ to podocytes incubated with palmitate in Ca^2+^-free KRB solution for 90 min. Palmitate-treated cells had pronounced [Ca^2+^]_*i*_ increase upon Ca^2+^ addition via SOCE compared to BSA-treated cells (Figures 4n and o).

**Figure 5:**
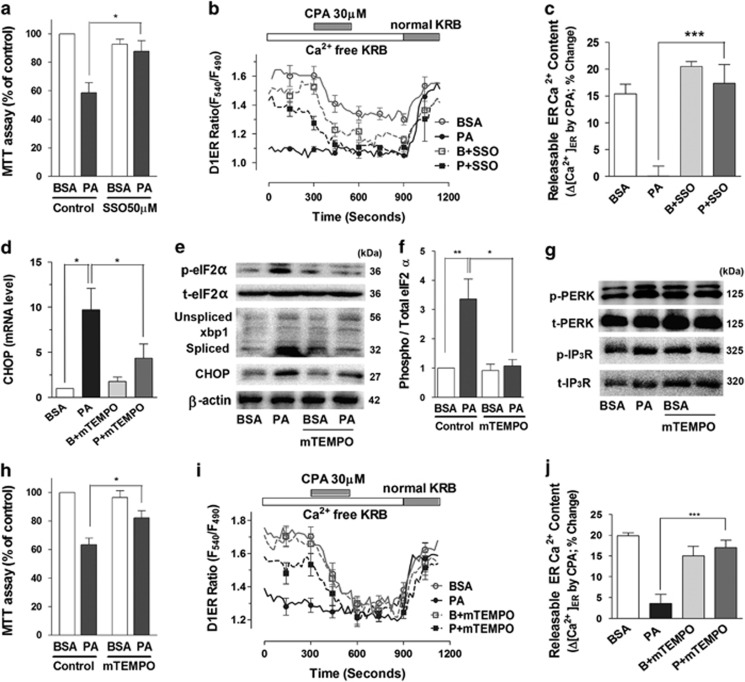
Palmitate-induced ER Ca^2+^ depletion and cytotoxicity were attenuated by a FAT/CD36 inhibitor and a mitochondrial antioxidant. (**a**) Sulfosuccinimidyl oleate (SSO), a FAT/CD36 inhibitor, was used to pretreat podocytes for 30 min before BSA or palmitate (PA, 300 *μ*M) incubation (for 24 h), and cytotoxicity was estimated by using an MTT assay (*N*=5). (**b**, **c**) Luminal Ca^2+^ content in ER ([Ca^2+^]_ER_) was measured in D1ER-transfected podocytes incubated with BSA or PA in Ca^2+^-free KRB solution for 90 min (*N*=5). (**d**–**j**) A mitochondrial antioxidant, mitoTEMPO (mTEMPO; 100 nM), was pretreated to podocytes for 30 min before BSA or PA incubation. The mRNA (**d**) and protein (**e**–**g**) levels of ER stress-related proteins were analyzed, and cytotoxicity induced by palmitate ((**h**), *N*=11) were estimated. Effects of mTEMPO pretreatment on [Ca^2+^]_ER_ levels were analyzed in D1ER-transfected podocytes incubated with BSA or PA upon cyclopiazonic acid (CPA) application ((**i**, **j**), *N*=13–15). Data are presented as mean ± S.E.M. *, ** and *** denote *P*<0.05, *P*<0.01 and *P*<0.001, respectively

FAT/CD36 is a fatty-acid transporter also known as scavenger receptor, which mediates adverse effects of saturated FFA.^37^ To test whether FAT/CD36 was required for palmitate-induced [Ca^2+^]_ER_ loss and cytotoxicity, we pretreated podocytes with the inhibitor, sulfosuccinimidyl oleate (SSO), 30 min before palmitate incubation (Figures 5a–c). SSO (50 *μ*M) abrogated palmitate-induced cytotoxicity compared with palmitate alone. Consistently, SSO restored the D1ER ratio and recovered the CPA-induced ER Ca^2+^ release. These results show that inhibition of FAT/CD36 prevents palmitate-induced depletion of ER Ca^2+^.

To investigate the role of palmitate-induced ROS on ER Ca^2+^ depletion and ER stress, we preincubated podocytes with the mitochondrial antioxidant, mitoTEMPO (100 nM). Palmitate (300 *μ*M)-induced cytotoxicity was significantly attenuated by preincubation with mitoTEMPO (Figure 5g). Transcriptional upregulation of CHOP by palmitate (300 *μ*M) was also inhibited by mitoTEMPO (Figure 5d). Palmitate increased the phosphorylation of eukaryotic translation initiation factor 2*α* (eIF2*α*), PERK and IP_3_R as well as the protein levels of CHOP and spliced but not unspliced xbp1. All these palmitate-induced changes were prevented by mitoTEMPO-mediated ROS scavenging (Figures 5e–g). The reduction of releasable ER Ca^2+^ and refilling of ER Ca^2+^ stores by palmitate were prevented by pretreatment with mitoTEMPO (Figures 5i and j). These data support our hypothesis that the oxidative stress originating from mitochondria participates in ER Ca^2+^ depletion and ER stress, which is instrumental in palmitate-elicited cytotoxicity.

**Figure 6:**
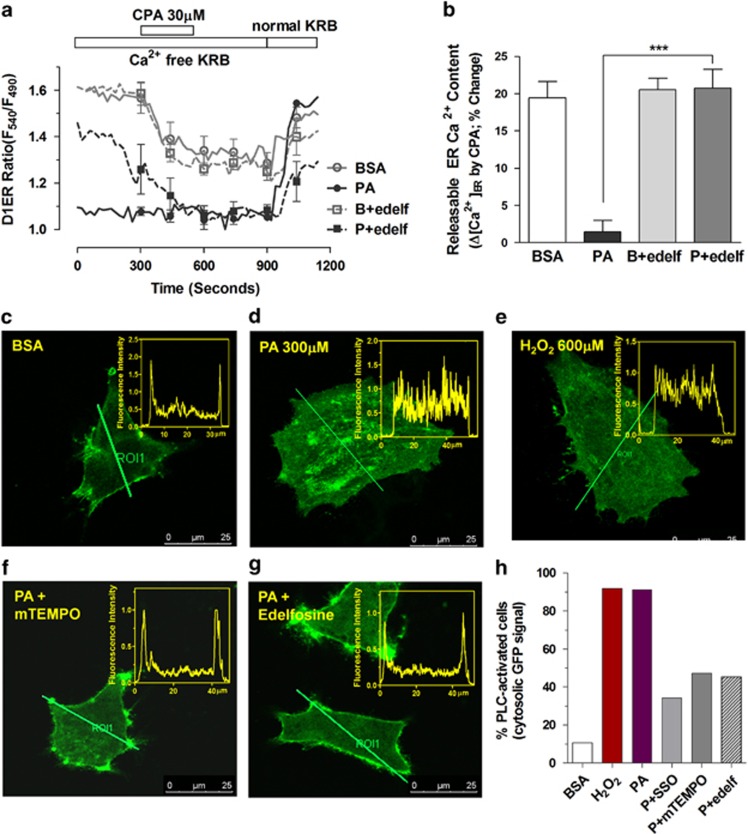
Inhibition of phospholipase C prevented intracellular IP_3_ generation and loss of the ER Ca^2+^ pool in palmitate-treated podocytes. (**a**, **b**) Changes in luminal Ca^2+^ content in ER ([Ca^2+^]_ER_) upon cyclopiazonic acid (CPA) application were measured in D1ER-transfected podocytes. Edelfosine (edelf; 10 *μ*M), a phospholipase C (PLC) inhibitor, was used to pretreat cells for 30 min before BSA or palmitate (PA, 300 *μ*M) incubation in Ca^2+^-free KRB solution for 90 min (*N*=4). (**c**–**h**) To observe IP_3_ generation from PIP_2_, a plasmid encoding the pleckstrin homology domain of phospholipase C*δ* (PLC*δ*-PH)-green fluorescence protein (GFP) was transfected into podocytes. (**c**–**g**) Podocytes were incubated with BSA, PA or H_2_O_2_ in Ca^2+^-free KRB solution for 90 min and GFP distribution was observed using a confocal microscope. Edelfosine (**f**) or a mitochondrial antioxidant, mitoTEMPO (mTEMPO; 100 nM) (**g**) was used to pretreat cells for 30 min before BSA or PA incubation. Line analyses of the fluorescence intensity for each picture are shown on the right. The percentage of cells showing PLCδ-PH-GFP translocation from the plasma membrane to the cytosol reflects PLC activation and IP_3_ synthesis. (**h**) Data are presented as mean±S.E.M. *** denote *P*<0.001

We applied the PLC inhibitor edelfosine to test whether PLC activation is involved in palmitate-induced ER Ca^2+^ release. Pretreatment with edelfosine completely restored the CPA-releasable ER Ca^2+^ pool in podocytes treated with palmitate (Figures 6a and b).

To directly visualize PLC activation in palmitate-treated podocytes, we overexpressed the pleckstrin homology (PH) domain of PLC-*δ* fused to green fluorescent protein (PH-GFP), which can bind phosphatidylinositol 4,5-bisphosphate (PIP_2_) in plasmalemmal lipid vesicles and also IP_3_ in the cytosol (Figures 6c–h).^38^ The activation of PLC elicits marked redistribution of the PH-GFP signal from the plasma membrane to the cytosol. PH-GFP fluorescence is concentrated in subplasmalemmal areas of control podocytes. This is conspicuously different from that of palmitate-treated cells, most of which have uniformly distributed GFP signal indicating re-localization with IP_3_ to the cytosol. The redistribution of the PH-GFP by palmitate was completely reproduced by H_2_O_2_ treatment, confirming oxidative stress-mediated PLC activation. Pretreatment with SSO to inhibit FAT/CD36 markedly augmented the proportion of palmitate-treated cells with subplasmalemmal GFP signal. PLC activation was also blocked by mitoTEMPO and the PLC inhibitor edelfosine in palmitate-treated cells. Based on the above results, we suggest that activation of PLC triggered by mitochondrial ROS is a critical step resulting in palmitate-induced ER Ca^2+^ depletion in podocytes.

**Figure 7:**
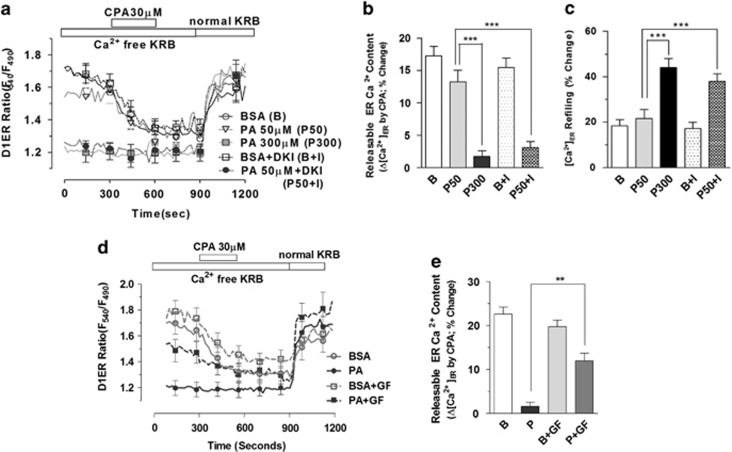
Diacylglycerol and protein kinase C are involved in ER Ca^2+^ depletion in palmitate-treated podocytes. Changes in luminal Ca^2+^ content in ER ([Ca^2+^]_ER_) upon cyclopiazonic acid (CPA) application were measured in D1ER-transfected podocytes incubated with BSA or PA (300 *μ*M) in Ca^2+^-free KRB solution for 90 min. (**a**–**c**) A diacylglycerol (DAG) kinase inhibitor, R59022 (DKI) was used to pretreat cells for 30 min before 50 *μ*M PA incubation, and CPA-induced reduction of [Ca^2+^]_ER_ (**b**) and ER Ca^2+^ refilling by 1.8 mM extracellular Ca^2+^ (**c**) were compared among different groups (*N*=13–24). (**d**) An inhibitor of protein kinase C, GF109203X was used to pretreat cells for 30 min before BSA or PA incubation, and CPA-induced reduction of [Ca^2+^]_ER_ was analyzed (*N*=19). Data are presented as mean±S.E.M. ** and *** denote *P*<0.01 and *P*<0.001, respectively

Using PIP_2_ as a substrate, PLC also generates diacylglycerol (DAG), which activates protein kinase C (PKC).^39^ DAG is further converted into phosphatidic acid by DAG kinase. Consequently, inhibition of DAG kinase increases DAG content and PKC activity.^40^ To evaluate the role of DAG in ER Ca^2+^ depletion, we co-incubated podocytes with R59022, a DAG kinase inhibitor (DKI), and palmitate (Figures 7a–c). ER Ca^2+^ homeostasis was not affected by low concentrations of palmitate (50 *μ*M) alone. However, the DKI (10 *μ*M) enabled palmitate (50 *μ*M) to deplete the ER Ca^2+^ store similar to the effect of high palmitate concentration (300 *μ*M). ER Ca^2+^ refilling was also strongly stimulated by the DKI in the presence of 50 *μ*M palmitate when compared with the same concentration of palmitate alone.

The role of PKC, which is a downstream effector of DAG, on palmitate-induced ER Ca^2+^ depletion was investigated using GF109203X, a nonspecific PKC inhibitor. GF109203X (5 *μ*M) partially restored the palmitate (300 *μ*M)-induced loss of releasable ER Ca^2+^ content (Figures 7d and e). These results show that activation of the PLC-DAG-PKC pathway is, at least in part, involved in ER Ca^2+^ depletion induced by palmitate.

**Figure 8:**
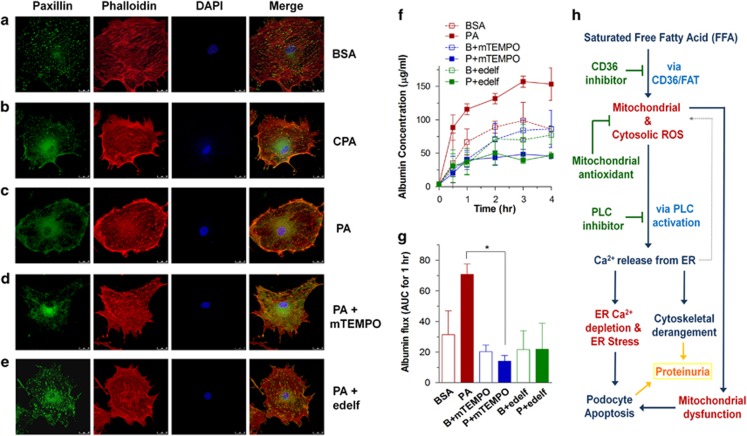
Alterations in the actin cytoskeleton and albumin permeability in palmitate-treated podocytes. Differentiated podocytes cultured on coverslips were incubated in Ca^2+^-free KRB solution containing BSA (**a**) or palmitate (PA; 300 *μ*M) (**c**) for 90 min, or cyclopiazonic acid (CPA; 15 *μ*M) for 15 min (**b**). A mitochondrial antioxidant, mitoTEMPO (mTEMPO; 100 nM) (**d**) or a phospholipase C (PLC) inhibitor, edelfosine (edelf; 10 *μ*M) (**e**), were used to pretreat podocytes for 15 min before PA incubation. Actin filaments were stained with phalloidin (red) and focal adhesions were detected by paxillin (green) staining. (**f**, **g**) The effects of BSA or PA on albumin permeability were estimated using transwell chambers with FITC-labeled BSA. (**h**) Proposed hypothetical model for palmitate-induced toxicity

Alteration of the cytoskeletal structure in podocytes impairs barrier function and protein filtration. To observe cytoskeletal rearrangements, fluorescence-labeled phalloidin and immunostaining of paxillin were used for visualizing actin fibers and focal adhesions, respectively (Figures 8a–e). BSA-treated differentiated podocytes have well-organized stress fiber bundles in the cytosol extended into the cell periphery. Paxillin was found at focal adhesions at the tip of actin fibers. Similar to the phenotype of CPA-treated podocytes, palmitate elicits cortical rearrangement of actin fibers with reduced focal adhesion points. Palmitate-induced changes were prevented by pretreatement with mitoTEMPO and edelfosine.

As a read-out of podocyte function, we measured the permeability of confluent podocyte layers to FITC-labeled BSA using transwell chambers. Palmitate treatment markedly increased albumin permeation due to defective barrier function of the podocytes (Figure 8f). Consistent with the restoration of cytoskeletal structure, high albumin permeability caused by palmitate was reduced by either mitoTEMPO or edelfosine (Figure 8g). These findings suggest that the pathogenic impact of palmitate on filtration barrier function in podocytes is closely related to ER Ca^2+^ release mediated by oxidative stress and PLC activation.

## Discussion

High serum levels of saturated FFA in diabetic patients have a detrimental effect on insulin secreting *β*-cells and target cells of the hormone. Previous studies have documented palmitate-induced pathologic changes in human and mouse cultured podocytes, including insulin resistance, ER stress and apoptosis.^14, 26^ We report in this study on mouse podocytes that palmitate, but not oleate, (1) induced mitochondrial and cytosolic ROS production and apoptotic cell death, (2) impaired mitochondrial energy metabolism (depolarization of the ΔΨm leading to defective ATP synthesis), (3) caused alterations of mitochondrial and ER morphology, (4) decreased the ER Ca^2+^ concentration, (5) elicited ER stress responses and (6) caused cytoskeletal rearrangement and albumin permeability in podocyte monolayers. Most of these palmitate-induced changes were prevented by co-incubation with oleate, inhibition of CD36/FAT, inhibition of PLC or scavenging of mitochondrial ROS.

Oxidative stress affects protein-folding capacity of the ER and activates the unfolded protein response.^21^ Persistent exposure to ROS initiates the pro-apoptotic pathway of ER stress and caspase activation.^25^ Both oxidative stress and ER stress may increase Ca^2+^ release from the ER, which further aggravates the ER function and cell fate.^23^ A key finding of this study was the pronounced palmitate-induced depletion of the ER Ca^2+^ pool. This was monitored directly by measuring luminal ER Ca^2+^ levels.^34^ The loss of ER Ca^2+^ stores by palmitate was complete, as shown by the absence of a response upon inhibition of the ER Ca^2+^ ATPase (SERCA). In addition, cytosolic Ca^2+^ rises upon stimulation of ER Ca^2+^ release were abolished in palmitate-treated podocytes. As a consequence of the pronounced depletion of ER Ca^2+^, we also observed STIM1 oligomerization following palmitate treatment. STIM1 oligomerization is part of the mechanism leading to store-operated calcium entry (SOCE), an attempt of palmitate-treated cells to replenish their ER calcium content.^36^ Downstream consequences of this impairment of ER Ca^2+^ homeostasis were dilatation of the ER lumen and ER stress responses. The reduced ER Ca^2+^ levels are causally related to the upregulation of ER stress-associated proteins, including CHOP and the initiation of apoptosis.

The present study furthers our understanding of the molecular mechanisms linking mitochondrial ROS production to ER stress. Activation of PLC has a central role in this process. This was demonstrated using a PH domain containing fluorescent protein, which allows visualization of PLC activation and PIP_2_ hydrolysis.^38^ Palmitate-induced PIP_2_ hydrolysis was prevented by a mitochondrial antioxidant similar to the effect of a PLC inhibitor. ER Ca^2+^ depletion was also abolished by the mitochondrial antioxidant as well as by PLC inhibition in the podocytes. Relevant in this context, a recent study proposed that ROS generated from NADPH oxidase 2 directly induces activation of PLC-*γ* via inhibition of DAG kinase in a lung ischemia–reperfusion model.^41^ Based on our result, we suggest that parallel activations of IP_3_ and DAG-PKC by PLC may contribute to palmitate-induced ER Ca^2+^ depletion and ER stress. Further clarification of the underlying molecular mechanisms is necessary.

In addition to ER Ca^2+^ release, SOCE through plasmalemmal orai channels could increase cytosolic as well as mitochondrial matrix Ca^2+^ concentrations. Ca^2+^ overload in the matrix facilitates mitochondrial superoxide generation.^42^ This mitochondrial ROS may further accelerate ER Ca^2+^ release.^23^ Such a positive feedback loop established from oxidative stress and ER-mitochondrial Ca^2+^ transfer compromises mitochondrial function and elicits PT. The observed reduction of the electrical gradient across the inner mitochondrial membrane, defective ATP synthesis and podocyte apoptosis could be the result of this type of mitochondrial oxidative stress. The importance of ER Ca^2+^ release in the promotion of cell death has already been demonstrated. The overexpression of SERCA promotes oxidative stress-mediated mitochondrial PT pore opening and apoptosis.^43^

The here shown tight interaction between mitochondrial and ER ROS and calcium-handling occurs in the regions of close proximity between mitochondria and the ER.^44^ These contact sites, mitochondria-associated ER membranes (MAMs), are a signaling platform and essential for normal ER and mitochondrial function.^45^ Loss of functional MAMs has been associated with hepatic insulin resistance in type 2 diabetes.^46^ In contrast to these findings, higher IP_3_ receptor expression and enlarged MAMs were reported in diabetic mice or control mice on a high-fat diet.^42^ The reason for these conflicting results remains to be elucidated.

To maintain proper glomerular filtration barrier function, the structure of the actin cytoskeleton in podocytes should adapt to environmental changes to prevent permeability to large molecules. Particularly, Ca^2+^-dependent remodeling of the actin cytoskeleton is essential for the maintenance of the glomerular slit diaphragm structure.^47^ Sustained Ca^2+^ elevation leads to the loss of stress fibers and foot effacement in podocytes, which causes glomerulosclerosis in mice.^48^ Palmitate-induced cytoskeleton rearrangement built cortical actin bundles (Figure 8). Blocking of either mitochondrial ROS or PLC signaling prevented actin cytoskeleton remodeling as well as albumin permeability of confluent podocyte monolayers. Our study provides evidence that Ca^2+^ release from the ER via oxidative stress and PLC activation could be responsible for pathologic actin remodeling and albumin permeability. In addition to podocyte apoptosis, alterations in the cytoskeleton may actively participate in the proteinuric phenotype induced by saturated FFA in DN.

Based on these results, we suggest several targets to achieve protection from glomerular injury and proteinuria as well as cellular damage in other tissues caused by high plasma levels of saturated FFA. First, scavenging the mitochondrial ROS could be an effective strategy to prevent the vicious cycle of oxidative stress and disturbance of organellar Ca^2+^. Mitochondrial antioxidants counteracted palmitate-mediated ER stress and cell death. Second, palmitate-induced pathogenic alterations were consistently alleviated by a mono-unsaturated FFA. Third, reducing PLC signaling, lowering downstream signaling pathways linked to IP_3_, DAG and PKC, could attenuate ER Ca^2+^ depletion and ER stress, as shown here using a PLC inhibitor. Last, but not least, pharmacological inhibition of FAT/CD36 efficiently reduces entry of FFA and its subsequent toxicity. In conclusion, we propose a model for the mechanism of saturated FFA-induced cytotoxicity in podocytes, which provides evidence for the close interaction between oxidative stress and Ca^2+^ homeostasis in mitochondria and the ER (Figure 8h). Our work contributes to the elucidation of high-fat-induced glomerular pathophysiology and paves the way for therapeutic approaches to various disease conditions with inherent ER stress and mitochondrial dysfunction.

## Materials and Methods

### Cell culture and drugs

Immortalized mouse podocyte cell line was a kind gift from Professor Peter Mundel (Harvard Medical School, Charlestown, MA, USA). These cells were grown on collagen I (Catalog # A10483-01, Life Technologies Corporation, Grand Island, NY, USA) coated dishes with low glucose (5.5 mM) Dulbecco's modified Eagles medium supplemented with 5% fetal bovine serum, 100 U/ml penicillin and 100 *μ*g/ml streptomycin at two different temperatures.^49, 50^ At 33 °C, cells are allowed to proliferate (permissive condition) in the presence of 20 U/ml mouse recombinant IFN-*γ* (R & D Systems, Minneapolis, MN, USA). For the induction of differentiation (non-permissive condition), podocytes were thermo-shifted to 37 °C in the absence of IFN-*γ* for 14 days. Expression of synaptopodin, a specific marker for podocyte differentiation, was gradually increased during culture in non-permissive conditions (37 °C), whereas it was not detected in podocytes maintained under permissive conditions (33 °C with interferon-*γ*).

Palmitate and oleate were prepared by conjugation with fatty-acid-free BSA. First, sodium palmitate or sodium oleate (100 mM) was dissolved in autoclaved distilled water (DW) at 70 °C for 30 min. BSA (10%) was also dissolved in autoclaved DW at 55 °C for 30 min and filtered. Dissolved palmitate or oleate solution was added dropwise into the BSA solution and stored as a stock (10 mM) at −80 °C until use. KRB solution contained (in mM) 135 NaCl, 3.6 KCl, 2 NaHCO_3_, 0.5 NaH_2_PO_4_, 0.5 MgSO_4_, 1.5 CaCl_2_, 10 HEPES, 5.5 glucose (pH 7.4, 318 mOsm/kg H_2_O). 2′,7′-Dichlorofluorescein diacetate DCF-DA, mitoSox, JC-1 and TMRM were purchased from Molecular Probes (Invitrogen, Grand Island, NY, USA). SSO was purchased from Santa Cruz Biotechnology (Santa Cruz, CA, USA), and mitoTEMPO was purchased from Enzo Life Sciences (Enzo Life Sciences, Inc., Farmingdale, NY, USA). Sodium palmitate, sodium oleate, fatty-acid-free BSA, MTT, 4′,6′-diamidino-2-phenylindole (DAPI), edelfosine, DKI R59022, GF109203X and other drugs were purchased from Sigma (St. Louis, MO, USA).

### Assays for apoptotic cell death

Cytotoxicity was estimated by MTT colorimetric assay. Differentiated podocytes seeded onto a 96-well plate were incubated with MTT (50 *μ*g/well) for 2 h. The supernatant was removed and 100 *μ*l dimethylsulfoxide was subsequently added to each well. After shaking the plate, the absorbances of each well at 570 and 630 nm were measured, and subtracted by a microplate ELISA reader (Molecular Devices, Sunnyvale, CA, USA).

Apoptosis of podocyte was detected by nuclear staining using DAPI. Podocytes were grown on collagen-coated coverslips and maintained for 14 days in non-permissive conditions. Before fixation, the medium was discarded and cells were carefully washed three times with phosphate-buffered saline (PBS). The cells were fixed using 4% paraformaldehyde in PBS for 15 min at 37 °C, followed by washing with PBS for three times. DAPI (1 μg/ml, 5 min) was used for nuclear staining. Cells fixed on coverslips were washed out three times with PBS and mounted on a glass slide using Vectashield mounting solution (Vector Laboratories, Inc., Burlingame, CA, USA). Apoptotic cells displayed characteristic features including chromatin condensation and nuclear fragmentation resulting from activation of endogenous nucleases, and were counted under an epifluorescence microscope (excitation at 358 nm and emission at 461 nm).

Apoptotic internucleosomal DNA fragmentation was quantitatively assayed by antibody-mediated capture of cytoplasmic oligonucleosome-associated histone-DNA complexes (Cell Death Detection ELISA Plus kit; Roche Molecular Biochemicals, Mannheim, Germany). Differentiated podocytes cultured in 24-well plates (5 × 10^4^ cells/well) were resuspended in 200 *μ*l lysis buffer supplied by the manufacturer, and incubated for 30 min at room temperature. After pelleting nuclei by centrifugation (200 × *g*, 10 min), 20 *μ*l of supernatant (cytoplasmic fraction) was used in the enzyme-linked immunosorbent assay (ELISA), following the manufacturer's standard protocol. Finally, the absorbances at 405 and 490 nm (reference wavelength), upon incubating with a peroxidase substrate for 5 min, were determined with a microplate ELISA reader (Molecular Devices).

### Measurement of cytosolic and mitochondrial ROS generation

Cytosolic ROS generation was measured using CM-H_2_DCF-DA (2′-7′ dichlorofluorescin diacetate) (Molecular Probes, Eugene, OR, USA). Differentiated podocytes on 12-mm coverslips were loaded with 5 μM CM-H_2_DCF-DA for 20 min at 37 °C, and excess dye was then washed out using KRB solution. Fluorescence intensity was measured using an inverted microscope (IX81, Olympus, Tokyo, Japan) equipped with an array laser Nipkow spinning disk (CSU10, Yokogawa Electric Corporation, Tokyo, Japan). The wavelengths for the measurement of DCF fluorescence were 490 nm for excitation and 535 nm for emission and were analyzed using Metamorph 6.1 software (Molecular Devices).

Mitochondrial superoxide generation was detected using mitoSox (Molecular Probes), a red fluorescent dye localized to mitochondria. Once it enters the mitochondria, mitoSox is specifically oxidized by superoxide and exhibits red fluorescence. MitoSox (5 *μ*M) was used to load the differentiated podocytes cultured on 12 mm coverslips for 14 days at 37 °C. Cells were washed twice with KRB solution and the red fluorescent intensity from podocytes was measured by using the Nipkow spinning disk confocal microscopic system with wavelengths of 514 and 560 nm for excitation and emission, respectively.

### Assays for ΔΨm and ATP generation

Mitochondrial membrane potential of podocytes was measured using the lipophilic cationic dye 5,5′,6,6′-tetrachloro-1,19,3,39-tetraethylbenzimidazolyl-carbocyanine iodide (JC-1) (Molecular Probes). JC-1 monomers enter into mitochondria based on ΔΨm and form J-aggregates inside the mitochondria transmitting red fluorescence (excitation/emission wavelength: 540/590 nm), whereas the rest of the monomers transmit green fluorescence (excitation/emission wavelength: 490/540 nm). The ratio of red/green fluorescence is used as an indicator of ΔΨm. For this experiment, differentiated podocytes were grown on 96-well flat, clear bottom, black-walled polystyrene TC-treated microplates (Corning Incorporated, Corning, NY, USA). Cells were washed twice with KRB solution after loading with JC-1 (300 nM) for 40 min and the fluorescence was recorded by a fluorescence microplate reader (Flexstation II, Molecular Devices), as described previously.^29^

As an alternative method to measure the ΔΨm, podocytes seeded on collagen I-coated coverslips were loaded with 8 nM TMRM for 20 min. Cells were placed on the inverted microscope and perfused with KRB solution containing 8 nM TMRM. Fluorescence images with 514 nm excitation and 560 nm emission were recorded with the Nipkow spinning disk confocal microscopic system and analyzed using Metamorph 6.1 software.

The ATP content in cell lysates was measured using a bioluminescence assay, as described previously.^29^ Differentiated podocytes seeded onto 24-well plates (10^5^ cells/well) were preincubated with glucose-free KRB solution for 2 h prior to incubation with 5.5 mM normal KRB solution for 30 min. Then, supernatants were removed and 100 *μ*l lysis buffer supplied by the ATP measurement kit (Roche HS-II Biolumniscence kit, Mannheim, Germany) was added. After 5 min, cells from each well were scraped with pipette tips and transferred to a tube for centrifugation. The supernatant was put into a 96-well plate and the luminescence was measured using a luminometer (Synergy, BioTek Instruments, Winooski, VT, USA) after addition of the luciferase reagent (50 *μ*l) supplied with the kit. Measurement of the protein concentration of cell lysates was performed using the Bradford assay (BCA protein assay kit, Thermo Scientific, Rockford, IL, USA).

### Morphological analysis of mitochondria and ER

Adenovirus encoding mitochondrial-targeted cyan fluorescence protein (mitoCFP) were applied together with adenovirus expressing the reverse tetracyclin transactivator for 90–120 min at 37 °C, as described previously.^29^ After 48 h incubation, cells were fixed with 4% paraformaldehyde in PBS for 15 min. Fluorescence images for mitoCFP (excitation/emission: 440/490 nm) were obtained using a laser-scanning mode confocal microscopic system (TCS SPE, Leica Microsystems GmbH, Wetzlar, Germany).

To observe STIM1 localization in the ER, differentiated podocytes were transiently transfected with a plasmid encoding YFP-tagged STIM1 using the FuGENE HD transfection reagent (Promega, Madison, WI, USA) according to the manufacturer's instructions. Fluorescence images for YFP-STIM1 (excitation/emission: 488/535 nm) were obtained 48 h after transfection using the laser-scanning mode confocal microscope. DAPI (1 *μ*g/ml, 5 min) was used for nuclear staining.

To analyze changes in the electron microscopic ultrastructures of mitochondria and ER, differentiated podocytes were collected by centrifugation and treated with 2.5% glutaraldehyde fixative at 4 °C for 24 h. These samples were then rinsed with 0.1 M sodium cacodylate buffer and post-fixed with 1% osmium tetroxide in the same buffer for 2 h. After rinsing with 0.1 M cacodylate buffer, they were dehydrated for 15-min periods in increasing concentrations of ethanol (70, 80, 90, 95 and 100% v/v), exchanged through propylene oxide, and embedded in a mixture of epoxy resin. Sections were cut with a diamond knife on an ultramicrotome (ULTRACUT E, Reichert-Jung, Vienna, Austria) and were stained with 1% uranyl acetate for 14 min, followed by a lead staining reagent for 3 min. The sections were examined with a transmission electron microscope JEM 1200 EXII (JEOL, Tokyo, Japan).

### Quantitative real-time PCR and western blot anlaysis

Total RNA was isolated from cultured podocytes using the RNeasy kit (Qiagen GmbH, Hilden, Germany). The quantity and quality of RNA were assessed using a spectrophotometer set at 260 nm. First-strand cDNA was synthesized from 1 *μ*g of total RNA with a reverse transcription kit (Applied Bioscience, Foster City, CA, USA) using oligo-dT in a reaction volume of 20 *μ*l, according to the manufacturer's protocol. Parallel reactions without reverse transcriptase were performed to confirm the absence of genomic DNA amplification. Quantitative real-time PCR was performed using sequence-specific primers to measure the mRNA levels of BIP (GRP78; (+) ACT TGG GGA CCA CCT ATT CC, (−) AGG AGT GAA GGC CAC ATA CG), CHOP (C/EBP homologous protein; (+) CAC CAC ACC TGA AAG CAG AA, (−) ATC CTC ATA CCA GGC TTC CA), and the spliced ((+) TGA GTC CGC AGC AGG TG, (−) GCA GAC TCT GGG GAA GGA C) and unspliced ((+) AAG AAC ACG CTT GGG AAT GG, (−) CAT AGT CTG AGT GCT GCG GA) forms of xbp1 (X-box BIP 1). *β*-actin was used as reference control. For the analysis of the expression of each gene, experiments were conducted in triplicate in a real-time PCR system (7900HT, ABI prism, Foster city, CA, USA) using SYBR Green PCR Master Mix (Qiagen), as described previously.^34^

For total protein extraction, cells seeded on six-well plates were washed with ice-cold PBS and lysed with cold RIPA buffer (Thermo Fisher Scientific Inc.) containing protease inhibitor cocktail (Roche Diagnostics GmbH, Mannheim, Germany). The supernatants from lysates were electrophoresed on SDS-PAGE gels and then transferred to a polyvinylidene difluoride membrane (Merck Millipore, Billerica, MA, USA). The membrane was blocked in 5% BSA or 6% skim milk for 1h at room temperature, followed by incubation with primary antibody at 4 °C overnight. To estimate the ER stress response, the membrane was incubated with primary antibodies against phosphorylated and total eIF2*α* (1 : 1000, Catalog # sc-101670 and sc-133132, Santa Cruz Biotechnology), phosphorylated and total PERK (1 : 2000, Catalog # sc-32579 and sc-13073), xbp1 (1 : 1000, Catalog # sc-7160, Santa Cruz Biotechnology) and CHOP (1:500, Catalog # 612202, BioLegend, San Diego, CA, USA). Primary antibodies against phosphorylated and total IP_3_R (1 : 2000, Catalog # 8548 and # 3763, Cell signaling Technology, Danvers, MA, USA) and as a loading control, *β*-actin (1 : 5000, Catalog # ab6276, Abcam, Cambrige, UK) were used. Membranes were incubated for 1 h at room temperature in horseradish peroxidase-conjugated secondary antibody against either mouse or rabbit IgG (Catalog # 31450 and 31460, Thermo Scientific). The bands were visualized with an UVP Biospectrum-600 imaging system using enhanced chemiluminescence solution (Luminata Forte, Millipore Corporation, Billerica, MA, USA).

### Measurement of cytosolic and ER luminal Ca^2+^ concentrations

To measure cytosolic Ca^2+^ concentrations ([Ca^2+^]_*i*_), podocytes cultured on coverslips were loaded with fura-2/AM (5 *μ*M) in a dark room for 40–60 min at room temperature. Fura-2-loaded podocytes were then washed and transferred to a perfusion chamber on an inverted microscope (IX-70, Olympus). The cells were alternately excited at 340 and 380 nm by a monochromatic light source (LAMDA DG-4; Sutter, Novato, CA, USA), and fluorescence images were captured at 510 nm with an intensified CCD camera (Cascade; Roper, Duluth, GA, USA). The fluorescence intensity ratio from the two excitation wavelengths (F_340_/F_380_) reflecting [Ca^2+^]_*i*_ was estimated by using MetaFluor 6.1 software.

Luminal Ca^2+^ content in the ER ([Ca^2+^]_ER_) was measured with FRET-based cameleon protein probe D1ER, which allows ratiometric recording of emitted fluorescence from YFP (540 nm) and CFP (490 nm), as described previously.^34, 35^ The D1ER plasmid was transiently transfected into podocytes using FuGENE HD. Forty-eight hours after transfection, cells on coverslips were excited at 440 nm by using the Nipkow spinning disk confocal microscopic system, and the D1ER fluorescence intensity ratio, which reflects the [Ca^2+^]_ER_, derived from measuring two emission wavelengths (F_540_/F_490_), was determined using MetaFluor 6.1 software.

### Assay for PIP2 hydrolysis

Differentiated podocytes were transfected with plasmid encoding the PH domain of the PLC-δ-GFP fusion construct using FuGENE HD.^38^ The PH domain of PLC-*δ* can selectively bind PIP_2_ in plasmalemmal lipid vesicles and also IP_3_ in the cytosol, but only weakly bind other inositol phosphates and phosphatidylinositol 4-phosphate. Therefore, hydrolysis of PIP_2_ by endogenous PLCs and propagation of generated IP_3_ should shift the distribution of GFP fluorescence from the plasma membrane to the cytosol.^38^ After 48 h incubation, fluorescence images for GFP (excitation/emission: 488/535 nm) were obtained using a confocal microscope.

### Staining for actin cytoskeleton

Differentiated podocytes cultured on coverslips were washed twice with PBS and fixed with 4% paraformaldehyde in PBS for 15 min at 37 °C. Cells were incubated with Alexa549-phalloidin (Invitrogen) for 20 min at room temperature. Then, cells were permeabilized with 0.25% Triton X-100 and blocked with 1% BSA for 30 min. Incubation with primary antibody against paxillin (Abcam) was performed overnight at 4 °C, followed by incubation with secondary antibody (Life Technology). The cells were washed and stained with DAPI for 5 min. Fluorescence images were obtained using a confocal microscope.

### Albumin permeability assay

Differentiated podocytes were seeded on transwell bicameral chambers (0.4 μm pore; Corning, Cambridge, MA, USA) and transepithelial passage of FITC-labeled BSA (Sigma) was measured. Confluent differentiated podocytes were exposed to palmitate for 90 min in Ca^2+^-free KRB solution. In the upper compartment of transwell chambers, culture medium was replaced with 2 ml of FITC-BSA (250 μg/ml). From the lower compartment 100 μl samples were drawn after 0.5, 1, 2, 3 and 4 h of diffusion. The chamber was replenished with an equal volume fresh medium each time a sample was withdrawn. The fluorescence intensity of collected samples was measured using a fluorescence microplate reader (Flexstation II) at 450 nm, and the albumin concentrations in the samples were calculated using linear regression.

### Data analysis

Statistical comparisons between two groups of data were performed using two-tailed unpaired Student's *t-*tests. Multiple comparisons were determined using one-way analysis of variance followed by a *post hoc* test (Tukey's multiple comparison test). *P-*values <0.05 were considered significant.

## Abbreviations

FFA: free fatty acid
ER: endoplasmic reticulum
STIM1: stromal interaction molecule 1
PLC: phospholipase C
DAG: diacylglycerol
CKD: chronic kidney disease
DN: diabetic nephropathy
ROS: reactive oxygen species
PT: permeability transition
BIP: immunoglobulin heavy chain binding protein
IRE1: inositol-requiring protein 1
ATF6: activating transcription factor 6
PERK: PKR-like ER kinase
PDI: protein disulfide isomerase
ERO1: ER oxidoreductase
CHOP: C/EBP homology protein
IP_3_: inositol (1,4,5)-trisphosphate
SCD: stearoyl-CoA desaturase
ΔΨm: mitochondrial membrane potential
AR: aspect ratio
xbp1: X-box-binding protein 1
CPA: cyclopiazonic acid
SERCA: sarco/endoplasmic reticulum Ca^2+^-ATPase
SSO: sulfosuccinimidyl oleate
eIF2*α*: eukaryotic translation initiation factor 2*α*
PH: pleckstrin homology
PIP_2_: phosphatidylinositol 4,5-bisphosphate
PKC: protein kinase C
DKI: DAG kinase inhibitor
SOC: store-operated calcium
MAMs: mitochondria-associated ER membranes
